# Probabilistic Nearest Neighbors Classification

**DOI:** 10.3390/e26010039

**Published:** 2023-12-30

**Authors:** Bruno Fava, Paulo C. Marques F., Hedibert F. Lopes

**Affiliations:** 1 Department of Economics, Northwestern University, Evanston, IL 60208, USA; brunofava@u.northwestern.edu; 2 Insper Institute of Education and Research, Rua Quatá 300, São Paulo 04546-042, Brazil; hedibertfl@insper.edu.br

**Keywords:** probabilistic machine learning, nearest neighbors classification, NP-completeness

## Abstract

Analysis of the currently established Bayesian nearest neighbors classification model points to a connection between the computation of its normalizing constant and issues of NP-completeness. An alternative predictive model constructed by aggregating the predictive distributions of simpler nonlocal models is proposed, and analytic expressions for the normalizing constants of these nonlocal models are derived, ensuring polynomial time computation without approximations. Experiments with synthetic and real datasets showcase the predictive performance of the proposed predictive model.

## 1. Introduction

The now classic nearest neighbors classification algorithm, introduced in a 1951 technical report by Fix and Hodges (reprinted in [1]), marked one of the early successes of machine learning research. The basic idea is that, given some notion of proximity between pairs of observations, the class of a new sample unit is determined by majority voting among its *k* nearest neighbors in the training sample [2,3]. A natural question is whether it is possible to develop a probabilistic model that captures the essence of the mechanism contained in the classic nearest neighbors algorithm while adding proper uncertainty quantification of predictions made by the model. In a pioneering paper, Holmes and Adams [4] defined a probabilistic nearest neighbors model specifying a set of conditional distributions. A few years later, Cucala et al. [5] pointed out the incompatibility of the conditional distributions specified by Holmes and Adams, which do not define a proper joint model distribution. As an alternative, Cucala et al. developed their own nearest neighbors classification model, defining directly a proper, Boltzmann-like joint distribution. A major difficulty with the Cucala et al. model is the fact that its likelihood function involves a seemingly intractable normalizing constant. Consequently, in order to perform a Bayesian analysis of their model, the authors engaged in a *tour de force* of Monte Carlo techniques, with varied computational complexity and approximation quality.

In this paper, we introduce an alternative probabilistic nearest neighbors predictive model constructed from an aggregation of simpler models whose normalizing constants can be exactly summed in polynomial time. We begin by reviewing the Cucala et al. model in Section 2, showing by an elementary argument that the computational complexity of the exact summation of its normalizing constant is directly tied to the concept of NP-completeness [6]. The necessary concepts from the theory of computational complexity are briefly reviewed. Section 3 introduces a family of nonlocal models, whose joint distributions take into account only the interactions between each sample unit and its *r*-th nearest neighbor. For each nonlocal model, we derive an analytic expression for its normalizing constant, which can be computed exactly in polynomial time. The nonlocal models are combined in Section 4, yielding a predictive distribution that does not rely on costly Monte Carlo approximations. We run experiments with synthetic and real datasets, showing that our model achieves the predictive performance of the Cucala et al. model, with a more manageable computational cost. We present our conclusions in Section 5.

## 2. A Case of Intractable Normalization

This section sets the environment for the general classification problem discussed in this paper. We begin in Section 2.1 with the definition of the Cucala et al. Bayesian nearest neighbors classification model, whose normalizing constant requires an exponential number of operations for brute force calculation. We indicate the Monte Carlo techniques used by the authors to sample from the model posterior distribution, as well as the approximations made to circumvent the computational complexity issues. Section 2.2 reviews briefly the fundamental concepts of the theory of computational complexity, ending up with the characterization of NP-complete decision problems, which are considered intractable. Section 2.3 establishes by an elementary argument a connection between the summation of the normalizing constant appearing on the likelihood of the Cucala et al. model and one of the classical NP-complete problems. In a nutshell, we show that the availability of an algorithm to exactly compute the normalizing constant of the Cucala et al. model in polynomial time in an ordinary computer would imply that all so-called NP problems could also be solved in polynomial time under equivalent conditions.

### 2.1. The Cucala et al. Model

Suppose that we have a size *n* training sample such that for each sample unit we know the value of a vector $x_{i}\in R^{p}$ of predictor variables and a response variable $y_{i}$ belonging to a set of class labels L={1,2,⋯,L}. Some notion of proximity between training sample units is given in terms of the corresponding vectors of predictors. For example, we may use the Euclidean distance between the vectors of predictors of every pair of training sample units to establish a notion of neighborhood in the training sample. Given this neighborhood structure, let the brackets ${\left[i\right]}_{r}$ denote the index of the sample unit in the training sample that is the *r*-th nearest neighbor of the *i*-th sample unit, for i=1,⋯,n, and r=1,⋯,n−1.

Introducing the notations $x=(x_{1},\cdots ,x_{n})$ and $y=(y_{1},\cdots ,y_{n})$, the Cucala et al. model [5] is defined by the joint distribution

$$p\left(y|x,\beta ,k\right)=\frac{1}{Z(\beta ,k)}\exp\;\left(\frac{\beta }{k}\sum_{i=1}^{n}\sum_{r=1}^{k}I\left(y_{i}=y_{{\left[i\right]}_{r}}\right)\right),$$

in which β≥0 and k=1,⋯,n−1 are model parameters and I(·) denotes an indicator function. Notice that dependence on the predictors occurs through the neighborhood brackets ${\left[i\right]}_{r}$, which are determined by the $x_{i}$’s. The model normalizing constant is given by

$$Z\left(\beta ,k\right)=\sum_{y\in L^{n}}\exp\;\left(\frac{\beta }{k}\sum_{i=1}^{n}\sum_{r=1}^{k}I\left(y_{i}=y_{{\left[i\right]}_{r}}\right)\right).$$

From this definition, we see that direct (brute force) summation of Z(β,k) would involve an exponential number of operations ($O(L^{n})$). The much more subtle question about the possible existence of an algorithm that would allow us to exactly compute Z(β,k) in polynomial time is addressed in Section 2.3.

In their paper [5], the authors relied on a series of techniques to implement Markov Chain Monte Carlo (MCMC) frameworks in the presence of the seemingly intractable model normalizing constant Z(β,k). They developed solutions based on pseudo-likelihood [7], path sampling [8,9] (which essentially approximates Z(β,k) using a computationally intensive process, for each value of the pair (β,k) appearing in the iterations of the underlying MCMC procedure) and the Møller et al. auxiliary variable method [10]. Although there is currently no publicly available source code for further experimentation, at the end of Section 3.4 the authors report computation times ranging from twenty minutes to more than one week for the different methods using compiled code. We refer the reader to [5] for the technical details.

### 2.2. Computational Complexity

Informally, by a deterministic computer we mean a device or process that executes the instructions in a given algorithm one at a time in a single-threaded fashion. A decision problem is one whose computation ends with a “yes” or “no” output after a certain number of steps, which is referred to as the running time of the algorithm. The class of all decision problems, with the input size measured by a positive integer *n*, for which there exists an algorithm whose running time on a deterministic computer is bounded by a polynomial in *n*, is denoted by P. We think of P as the class of computationally “easy” or tractable decision problems. Notable P problems are the decision version of linear programming and the problem of determining if a number is prime.

A nondeterministic computer is an idealized device whose programs are allowed to branch the computation at each step into an *arbitrary* number of parallel threads. The class of nondeterministic polynomial (NP) decision problems contains all decision problems for which there is an algorithm or program that runs in polynomial time on a nondeterministic computer. An alternative and equivalent view is that NP problems are those decision problems whose solution is difficult to find but easy to verify. We think of NP problems as the class of computationally “hard” or intractable decision problems. Notable NP problems are the Boolean satisfiability (SAT) problem and the travelling salesman problem. Every problem in P is obviously in NP. In principle, for any NP problem, it could be possible to find an algorithm solving the problem in polynomial time on a deterministic computer. However, a proof for a single NP problem that there is no algorithm running on a deterministic computer that could solve it in polynomial time would establish that the classes P and NP are not equal. The problem of whether P is or is not equal to NP is the most famous open question of theoretical computer science.

Two given decision problems can be connected by the device of polynomial reduction. Informally, suppose that there is a subroutine that solves the first problem. We say that the first problem is polynomial time reducible to the second if both the time required to transform the first problem into the second and the number of times the subroutine is called are bounded by a polynomial in *n*.

In 1971, Stephen Cook [11] proved that all NP problems are polynomial-time-reducible to SAT, meaning that 1) no NP problem is harder than SAT and 2) a polynomial time algorithm that solves SAT on a deterministic computer would give a polynomial time algorithm solving every other NP problem on a deterministic computer, ultimately implying that P is equal to NP. In general terms, a problem is said to be NP-complete if it is NP and all other NP problems can be polynomial-time reduced to it, and SAT was the first ever problem proven to be NP-complete. In a sense, each NP-complete problem encodes the quintessence of intractability.

### 2.3. Z(β,k) and NP-Completeness

Let G=(V,E) be an undirected graph, in which *V* is a set of vertices and *E* is a set of edges $e=\{v,v^{\prime }\}$, with $v,v^{\prime }\in V$. Given a function $w:E\to Z_{+}$, we refer to w(e) as the weight of the edge e∈E. A cut of *G* is a partition of *V* into disjoint sets $V_{0}$ and $V_{1}$. The size of the cut is the sum of the weights of the edges in *E* with one endpoint in $V_{0}$ and one endpoint in $V_{1}$. The decision problem known as the *maximum cut* can be stated as follows: for a given integer *m*, is there a cut of *G* with size at least *m*? Karp [12] proved that the general maximum cut problem is NP-complete. In what follows, we point to an elementary link between the exact summation of the normalizing constant Z(β,k) of the Cucala et al. model and the decision of an associated maximum cut problem.

Without a loss of generality, suppose that we are dealing with a binary classification problem in which the response variable $y_{i}\in \left\{0,1\right\}$, for i=1,⋯,n. Define the n×n matrix $A=(a_{ij})$ by $a_{ij}=1$ if *j* is one of the *k* nearest neighbors of *i*, and $a_{ij}=0$ otherwise. Letting $B=\left(b_{ij}\right)=A+A^{⊤}$, this is the adjacency matrix of a weighted undirected graph *G*, whose vertices represent the training sample units, and the edges connecting these vertices may have weights zero, one, or two, based on whether the corresponding training sample units do not belong to each other’s *k*-neighborhoods, just one belongs to the other’s *k*-neighborhood, or both are part of each other’s *k*-neighborhoods, respectively. The double sum in the exponent of Z(β,k) can be rewritten as

$$T\left(y\right)=\sum_{i=1}^{n}\sum_{r=1}^{k}I\left(y_{i}=y_{{\left[i\right]}_{r}}\right)=\sum_{i,j=1}^{n}a_{ij}\;I\left(y_{i}=y_{j}\right)=\frac{1}{2}\sum_{i,j=1}^{n}b_{ij}\;I\left(y_{i}=y_{j}\right),$$

for every $y\in {\left\{0,1\right\}}^{n}$. Furthermore, each $y\in {\left\{0,1\right\}}^{n}$ corresponds to a cut of the graph *G* if we define the disjoint sets of vertices $V_{0}=\left\{i\in E:y_{i}=0\right\}$ and $V_{1}=\left\{i\in E:y_{i}=1\right\}$. The respective cut size is given by:

$$\mathrm{cut}\text{-}\mathrm{size}\left(y\right)=\frac{1}{2}\sum_{i,j=1}^{n}b_{ij}\;I\left(y_{i}\neq y_{j}\right).$$

Since for every $y\in {\left\{0,1\right\}}^{n}$ we have that

$$\sum_{i,j=1}^{n}b_{ij}=\sum_{i,j=1}^{n}b_{ij}\;I\left(y_{i}=y_{j}\right)+\sum_{i,j=1}^{n}b_{ij}\;I\left(y_{i}\neq y_{j}\right),$$

it follows that

$$\mathrm{cut}\text{-}\mathrm{size}\left(y\right)=\left(\frac{1}{2}\sum_{i,j=1}^{n}b_{ij}\right)-T\left(y\right).$$

**Figure 1:**
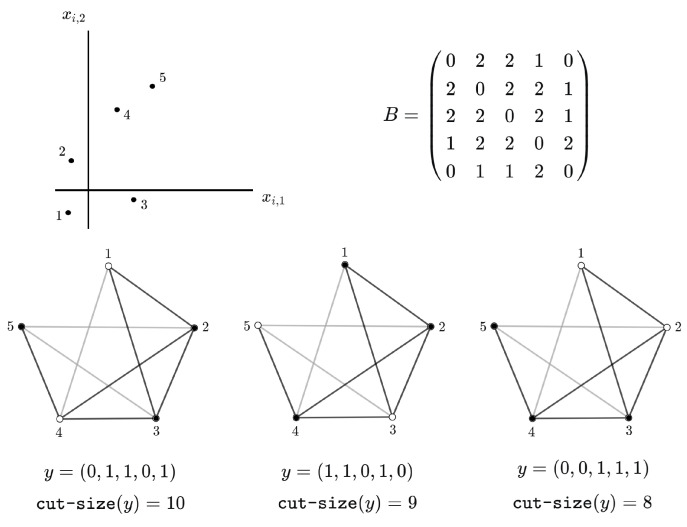
Weighted undirected nonplanar graphs associated with a specific neighborhood structure, determined by the three nearest neighbors according to Euclidean distance, and the sizes of three different cuts, obtained by summing the weights of all edges linking two vertices of different colors. On each graph, light gray and black edges have weights with values one and two, respectively, according to the adjacency matrix *B*. Black and white vertices correspond to class labels being equal to one and zero, respectively.

Figure 1 gives an example for a specific neighborhood structure involving the three nearest neighbors with respect to Euclidean distance.

By grouping each possible value of T(y) in the sum over $y\in {\left\{0,1\right\}}^{n}$ appearing in the definition of Z(β,k), we obtain the alternative polynomial representation

$$Z\left(\beta ,k\right)=\sum_{t=0}^{nk}d_{t}z^{t},$$

in which $z=e^{\beta /k}$ and $d_{t}=\sum_{y\in {\left\{0,1\right\}}^{n}}I\left(T\left(y\right)=t\right)$, for t=0,1,⋯,nk. Note that $d_{t}$ is the number of vectors $y\in {\left\{0,1\right\}}^{n}$ such that T(y)=t, and from (∗) we have that $d_{t}$ is also the number of possible cuts of the graph *G* with size $\left(\frac{1}{2}\sum_{i,j=1}^{n}b_{ij}\right)-t$.

Suppose that we have found a way to sum Z(β,k) in polynomial time on a deterministic computer, for every possible value of β and *k* and any specified neighborhood structure. By polynomial interpolation (see [13]), we would be able to compute the value of each coefficient $d_{t}$ in polynomial time, thus determining the number of cuts of *G* with all possible sizes, thereby solving any maximum cut decision problem associated with the graph *G*. In other words: the existence of a polynomial time algorithm to sum Z(β,k) *for an arbitrary neighborhood structure* on a deterministic computer would imply that P is equal to NP.

## 3. Nonlocal Models Are Tractable

This section introduces a family of models that are related to the Cucala et al. model but differ in two significant ways. First, making use of a physical analogy, while the likelihood function of the Cucala et al. model is such that each sampling unit “interacts” with all of its *k* nearest neighbors, for the models introduced in this section each sampling unit interacts only with its *r*-th nearest neighbor, for some r=1,⋯,n−1. Keeping up with the physical analogy, we say that we a have a family of nonlocal models (for the sake of simplicity, we are abusing terminology a little bit here, since the model with r=1 is perfectly “local”). Second, the nice fact about the nonlocal models is that their normalizing constants are tractable; the main result of this section being an explicit analytic expression for the normalizing constant of a nonlocal model that is computable in polynomial time. The purpose of these nonlocal models is to work as building blocks for our final aggregated probabilistic predictive model in Section 4.

For r=1,⋯,n−1, the likelihood of the *r*-th nonlocal model is defined as

$$p_{r}\left(y|x,\beta_{r}\right)=\frac{1}{Z_{r}\left(\beta_{r}\right)}\exp\;\left(\beta_{r}\sum_{i=1}^{n}I\left(y_{i}=y_{{\left[i\right]}_{r}}\right)\right),$$

in which the normalizing constant is given by

$$Z_{r}\left(\beta_{r}\right)=\sum_{y\in L^{n}}\exp\;\left(\beta_{r}\sum_{i=1}^{n}I\left(y_{i}=y_{{\left[i\right]}_{r}}\right)\right),$$

with parameter $\beta_{r}\geq 0$.

In line with what was pointed out in our discussion of the normalizing constant Z(β,k) of the Cucala et al. model, brute force computation of $Z_{r}\left(\beta_{r}\right)$ is also hopeless for the nonlocal models, requiring the summation of an exponential number of terms ($O(L^{n})$). However, the much simpler topology associated with the neighborhood structure of a nonlocal model can be exploited to give us a path to sum $Z_{r}\left(\beta_{r}\right)$ analytically, resulting in an expression that can be computed exactly in polynomial time on an ordinary computer.

Throughout the remainder of this section, our goal is to derive a tractable closed form for the normalizing constant $Z_{r}\left(\beta_{r}\right)$. For the *r*-th nonlocal model, consider the directed graph G=(V,E) representing the associated neighborhood structure of a given training sample. For i=1,⋯,n, each vertex i∈V corresponds to one training sample unit, and the existence of an oriented edge (i,j)∈E, represented pictorially by an arrow pointing from *i* to *j*, means that the *j*-th sample unit is the *r*-th nearest neighbor of the *i*-th sample unit.

**Figure 2:**
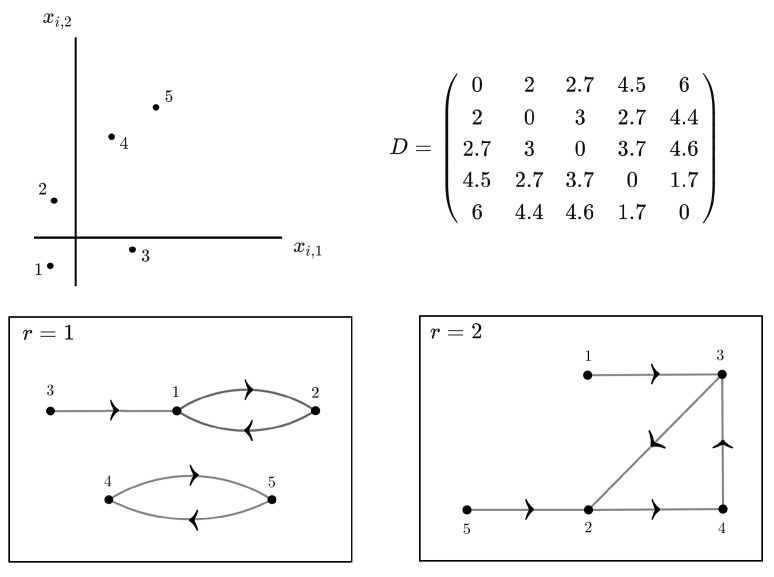
Distance matrix *D* and directed graphs showing the neighborhood structure for the nonlocal models with r=1 and r=2.

An example is given in Figure 2 for the nonlocal models with r=1 and r=2. We see that in general *G* can be decomposed into totally disconnected subgraphs $G^{\prime }=\left(V^{\prime },E^{\prime }\right),G^{\prime \prime }=\left(V^{\prime \prime },E^{\prime \prime }\right),\cdots$, meaning that vertices in one subgraph have no arrows pointing to vertices in the other subgraphs. If $V^{\prime }=\left\{i_{1},\cdots ,i_{k}\right\}$, we use the notation for the multiple sum

$$\sum_{\begin{array}{c} y_{i}=1\\ \;i\in V^{\prime } \end{array}}^{L}:=\sum_{y_{i_{1}}=1}^{L}\cdots \sum_{y_{i_{k}}=1}^{L}.$$

Since

$$\sum_{y\in L^{n}}:=\sum_{y_{1}=1}^{L}\sum_{y_{2}=1}^{L}\cdots \sum_{y_{n}=1}^{L},$$

the normalizing constant $Z_{r}\left(\beta_{r}\right)$ can be factored as a product of summations involving only the $y_{i}$’s associated with each subgraph:

$$Z_{r}\left(\beta_{r}\right)=\left(\;\sum_{\begin{array}{c} y_{i}=1\\ \;i\in V^{\prime } \end{array}}^{L}\exp\;\left(\beta_{r}\sum_{i=1}^{n}I\left(y_{i}=y_{{\left[i\right]}_{r}}\right)\right)\right)\times \left(\;\sum_{\begin{array}{c} y_{i}=1\\ \;i\in V^{\prime \prime } \end{array}}^{L}\exp\;\left(\beta_{r}\sum_{i=1}^{n}I\left(y_{i}=y_{{\left[i\right]}_{r}}\right)\right)\right)\times \cdots \;.$$

**Figure 3:**
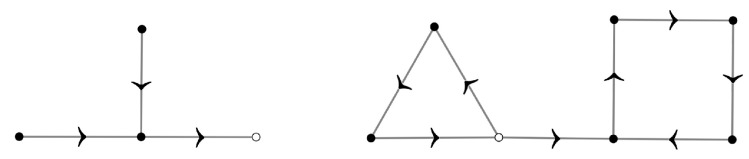
Two impossible directed graphs for a nonlocal model. On the left graph, the white vertex has outdegree equal to zero. On the right graph, the white vertex has outdegree equal to two. Both graphs contradict the fact that each training sample unit has exactly one *r*-th nearest neighbor. The conclusion is that in a directed graph describing the neighborhood structure of a nonlocal model each subgraph contains exactly one simple cycle.

For each subgraph, starting at some vertex and following the arrows pointing to each subsequent vertex, if we return to the first vertex after *m* steps, we say that the subgraph has a simple cycle of size *m*. The outdegree of a vertex is the number of arrows pointing from it to other vertices; the indegree of a vertex is defined analogously. Figure 3 depicts the fact that each subgraph has exactly one simple cycle: in a subgraph without simple cycles, there would be at least one vertex with outdegree equal to zero. Moreover, a subgraph with more than one simple cycle would have at least one vertex in one of the simple cycles pointing to a vertex in another simple cycle, implying that such a vertex would have outdegree equal to two. Both cases contradict the fact that every vertex of each subgraph has outdegree equal to one, since each sample unit has exactly one *r*-th nearest neighbor.

**Figure 4:**
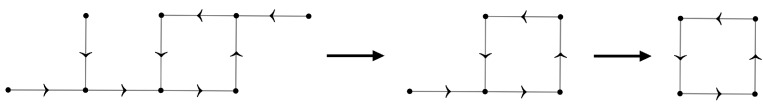
Example of the reduction process for the summation on each subgraph.

Figure 4 portrays the reduction process used to perform the summations for one subgraph. For each vertex with indegree equal to zero, we sum over the correspondent $y_{i}$ and remove the vertex from the graph. We repeat this process until we are left with a summation over the vertices forming the simple cycle. The summation for each vertex *i* with indegree equal to zero in this reduction process gives the factor

$$\sum_{y_{i}=1}^{L}\exp\left(\beta_{r}I\left(y_{i}=y_{{\left[i\right]}_{r}}\right)\right)=e^{\beta_{r}}+L-1,$$

because—and this is a crucial aspect of the reduction process—in this sum the indicator is equal to one just for a single term, and it is equal to zero for all the remaining L−1 terms, *whatever the value of* $y_{{\left[i\right]}_{r}}$. Summation over the vertices forming the simple cycle is performed as follows. Relabeling the indexes of the sample units if necessary, suppose that the vertices forming a simple cycle of size *m* are labeled as 1,2,⋯,m. Defining the matrix $S=(s_{a,b})$ by $s_{a,b}=\exp\left(\beta_{r}I\left(a=b\right)\right)$, we have

$$\begin{array}{cc} \; & \sum_{y_{1}=1}^{L}\sum_{y_{2}=1}^{L}\cdots \sum_{y_{m}=1}^{L}\exp\left(\beta_{r}I\left(y_{1}=y_{2}\right)\right)\times \exp\left(\beta_{r}I\left(y_{2}=y_{3}\right)\right)\times \cdots \times \exp\left(\beta_{r}I\left(y_{m}=y_{1}\right)\right)\\ \; & \;\;=\sum_{y_{1}=1}^{L}\sum_{y_{2}=1}^{L}\cdots \sum_{y_{m}=1}^{L}s_{y_{1},y_{2}}\times s_{y_{2},y_{3}}\times \cdots \times s_{y_{m},y_{1}}=\sum_{y_{1}=1}^{L}{\left(S^{m}\right)}_{y_{1},y_{1}}=\mathrm{Tr}\left(S^{m}\right). \end{array}$$

By the spectral decomposition [14], we have that $S=Q\Lambda Q^{⊤}$, with $QQ^{⊤}=Q^{⊤}Q=I$. Therefore, $S^{m}=Q\Lambda^{m}Q^{⊤}$, implying that $\mathrm{Tr}\left(S^{m}\right)=\mathrm{Tr}\left(\Lambda^{m}Q^{⊤}Q\right)=\mathrm{Tr}\left(\Lambda^{m}\right)=\sum_{\ell =1}^{L}\lambda_{\ell }^{m}$, in which we used the cyclic property of the trace, and the $\lambda_{\ell }$’s are the eigenvalues of *S*, which are easy to compute: the characteristic polynomial of *S* is

$$\det\left(S-\lambda I\right)={\left(e^{\beta_{r}}-1-\lambda \right)}^{L-1}\left(e^{\beta_{r}}+L-1-\lambda \right)=0,$$

yielding

$$\lambda_{1}=\lambda_{2}=\cdots =\lambda_{L-1}=e^{\beta_{r}}-1,\;\;\mathrm{and}\;\;\lambda_{L}=e^{\beta_{r}}+L-1.$$

For the *r*-th nonlocal model, let $c_{m}^{\left(r\right)}$ be the number of simple cycles of size *m*, considering all the associated subgraphs. Algorithm 1 shows how to compute $c_{m}^{\left(r\right)}$, for r=1,⋯,n−1 and m=2,⋯,n, in polynomial time. Taking into account all the subgraphs, and multiplying all the factors, we arrive at the final expression:

$$Z_{r}\left(\beta_{r}\right)={\left(e^{\beta_{r}}+L-1\right)}^{n-\sum_{m=2}^{n}m\times c_{m}^{\left(r\right)}}\times \prod_{m=2}^{n}{\left({\left(e^{\beta_{r}}+L-1\right)}^{m}+\left(L-1\right){\left(e^{\beta_{r}}-1\right)}^{m}\right)}^{c_{m}^{\left(r\right)}}.$$

**Algorithm 1** Count the occurrences of simple cycles of different sizes on the directed subgraphs representing the neighborhood structures of all nonlocal models**Require:** Neighborhood brackets $\left\{{\left[i\right]}_{r}:i=1,\cdots ,n;r=1,\cdots ,n-1\right\}$.  1:**function** count_simple_cycles($\left\{{\left[i\right]}_{r}:i=1,\cdots ,n;r=1,\cdots ,n-1\right\}$)2:    $c_{m}^{\left(r\right)}\leftarrow 0\mathrm{for}\left(r,m\right)\in \left\{1,\cdots ,n-1\right\}\times \left\{2,\cdots ,n\right\}$3:    **for** r←1ton−1 **do**4:        visited←∅5:        **for** j←1ton **do**6:           **next if** j∈visited7:           i←j8:           walk←emptystack9:           **while** i∉visited **do**10:               visited←visited∪{i}11:               pushiintowalk12:               $i\leftarrow {\left[i\right]}_{r}$13:           **end while**14:           m←115:           **while** walknotempty **do**16:               deletetopelementfromwalk17:               **if** topelementofwalk=i **then**18:                   $c_{m}^{\left(r\right)}\leftarrow c_{m}^{\left(r\right)}+1$19:                   **break**20:               **end if**21:               m←m+122:           **end while**23:        **end for**24:    **end for**25:    **return** $\left\{c_{m}^{\left(r\right)}:r=1,\cdots ,n-1;m=2,\cdots ,n\right\}$26:**end function**

## 4. Predictive Model

The nonlocal models developed in Section 3 will be the building blocks of our probabilistic nearest neighbors classification model. Introducing a hyperparameter k=1,⋯,n−1, the first *k* nonlocal models can be combined; the heuristic being that a “superposition” of nonlocal models could work as a model whose neighborhood structure takes into account the sets of *k* nearest neighbors of each sample unit. Section 4.1 explains the aggregation procedure leading to the predictive distribution of the combined model. The results in Section 4.2 showcase the computational cost and the predictive performance of the new model, examining the same synthetic and real datasets explored in [5].

### 4.1. Aggregating the Predictions of the Nonlocal Models

For the *r*-th nonlocal model, with r=1,⋯,n−1, using the information in the training sample and the analytic expression obtained for $Z_{r}\left(\beta_{r}\right)$ in Section 3, we construct an estimate ${\hat{\beta }}_{r}$ for the parameter $\beta_{r}$ by maximizing the corresponding likelihood function:

$${\hat{\beta }}_{r}=\arg\max_{\beta_{r}\geq 0}p_{r}\left(y|x,\beta_{r}\right)=\arg\max_{\beta_{r}\geq 0}\left(\frac{1}{Z_{r}\left(\beta_{r}\right)}\exp\;\left(\beta_{r}\sum_{i=1}^{n}I\left(y_{i}=y_{{\left[i\right]}_{r}}\right)\right)\right).$$

Using this estimate, we define the predictive distribution of the *r*-th nonlocal model as

$$\begin{array}{cc} p_{r}\left(y_{n+1}|x_{n+1},x,y\right) & :=p_{r}\left(y_{n+1}|x_{n+1},x,y,{\hat{\beta }}_{r}\right)\\ \; & \propto \exp\left({\hat{\beta }}_{r}\;\left(I\left(y_{n+1}=y_{{\left[n+1\right]}_{r}}\right)+\sum_{i=1}^{n}I\left(y_{i}=y_{n+1},{\left[i\right]}_{r}=n+1\right)\right)\right), \end{array}$$

with $\sum_{y_{n+1}=1}^{L}p_{r}\left(y_{n+1}|x_{n+1},x,y\right)=1$.

Finally, we aggregate the predictive distributions of the nonlocal models, introducing a hyperparameter k=1,⋯,n−1 and defining

$$p_{\left(k\right)}\left(y_{n+1}|x_{n+1},x,y\right):=\frac{1}{k}\sum_{r=1}^{k}p_{r}\left(y_{n+1}|x_{n+1},x,y\right).$$

The value of the hyperparameter *k* is chosen using leave-one-out cross validation [15] on the training sample. From now on, we refer to this predictive model as the probabilistic nearest neighbors classification model (pnnclass, for short).

An open source software library implementing the predictive model described in this section is publicly available at https://github.com/paulocmarquesf/pnnclass (access on 29 December 2023). It is an R [16] library, having internal routines written in C++ [17] with the help of Rcpp [18].

### 4.2. Experiments with Synthetic and Real Datasets

**Figure 5:**
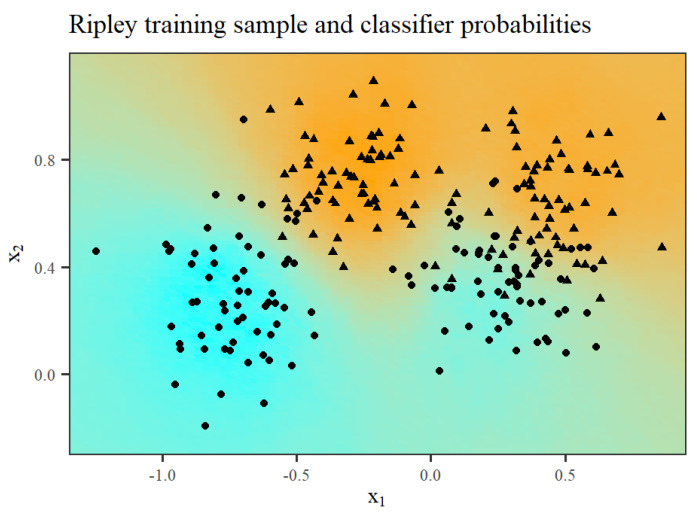
Training sample for the Ripley synthetic dataset. Circles and triangles indicate the two possible values of the response variable. The heatmap represents the probabilities of class “triangle” given by the predictive model pnnclass, ranging from pure cyan (probability zero) to pure orange (probability one).

**Figure 6:**
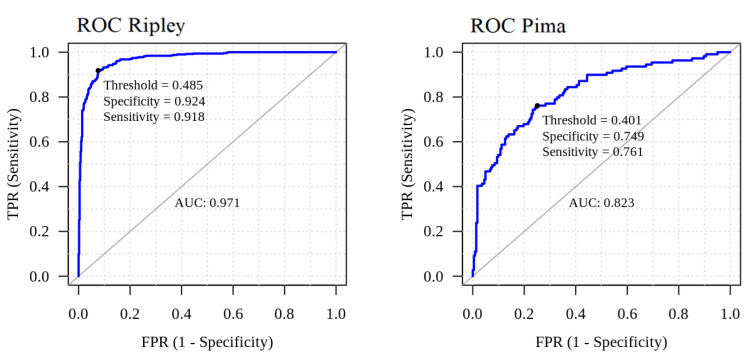
ROC curves for the Ripley and the Pima Indian datasets. Annotated summaries correspond to the probability threshold maximizing the sum of the classifier sensitivity and specificity.

The first example examined in [5] was the simulated dataset proposed by Ripley in his classic book [19]. It consists of training and testing samples with sizes 250 and 1000, respectively. The response variable is binary, and we have two generic real-valued predictor variables. The pnnclass predictive model achieves a testing error rate of 8.4%, which is the same as the best error rate reported in [5] for this dataset. Doing 100 replications, the median running time using the pnnclass library was 124.45 milliseconds, on a standard notebook with an Intel i7-11390H processor. Figure 5 shows the training sample and a heatmap representing the probabilities produced by the pnnclass predictive model. Figure 6 reports the classifier ROC curve obtained using the testing sample. The annotated summaries correspond to the probability threshold maximizing the sum of the classifier sensitivity and specificity.

The second example examined in [5] was the Pima Indian women diabetes dataset, available in the MASS R library [20], with training and test samples of sizes 200 and 332, respectively. In this dataset, for each sample unit we have the values of seven numerical predictors, and the response variable is again binary, indicating the result of the diabetes diagnosis for the corresponding sample unit. The pnnclass predictive model achieves a testing error rate of 21.99%, which is close to the best error rate reported in [5] for this dataset (20.9%). Performing 100 replications, the median running time using the pnnclass library was 59.04 milliseconds. The ROC curve and summaries for the Pima Indian dataset are given in Figure 6.

**Figure 7:**
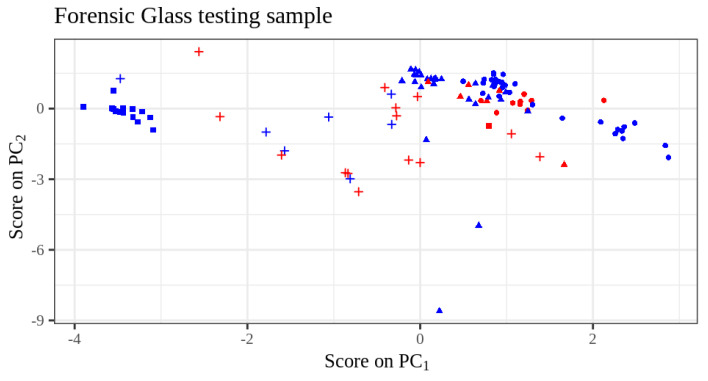
Testing sample scores on the first two principal components of the predictors in the Forensic Glass dataset. The different shapes indicate the four possible values of the response variable. The colors blue or red mean, respectively, that the corresponding testing sample units were classified correctly or incorrectly.

The third and final example considered in [5] was the multiclass Forensic Glass fragments dataset described in [19]. In this dataset, we have nine numerical predictors, and the response variable was coalesced into four classes. With randomly split training and testing samples of sizes 89 and 96, respectively, the pnnclass predictive model achieves a testing error rate of 28.04%, which is better than the error rate of 29% reported in [5] for this dataset. Performing 100 replications, the median running time using the pnnclass library was 9.15 milliseconds. Since in this dataset the predictor space is nine-dimensional, in Figure 7 we use Principal Component Analysis (PCA) as a visualization tool to depict the predictive performance on the the testing sample (PCA played no role in the modeling process). The confusion matrix for the Forensic Glass testing sample is given in Table 1. Table 2 gives more detailed summaries for the running times on the three datasets. Table 3 summarizes the predictive performance comparison between the pnnclass and the Cucala et al. models for the three datasets. For comparison, we added the test errors of a Random Forest [21,22] constructed by bootstrap aggregating 500 classification trees.

## 5. Concluding Remarks

This work can be seen as the construction of a probabilistic predictive model based on the concept of nearest neighbors, wherein we shifted the algorithmic complexity of the solution from an intractable class into the realm of polynomial time computation. It would be interesting to explore how the ideas discussed in this paper could be adapted to assist in the analysis of spatial data models or models with graph-type data in general, since these models may pose similar challenges related to the presence of intractable normalizing constants in the corresponding likelihood functions.

## Figures and Tables

**Table 1:** Confusion matrix for the Forensic Glass testing sample.

	Observed Class				
		0	1	2	3
Predicted class	0	29	2	0	5
	1	2	27	1	7
	2	0	1	14	2
	3	7	3	0	7

**Table 2:** Descriptive summaries for the running times in milliseconds of the pnnclass predictive model library based on 100 replications.

Dataset	Median (ms)	Min (ms)	Max (ms)
Ripley	124.45	118.83	182.34
Pima Indian	59.04	52.68	114.97
Forensic Glass	9.15	8.80	16.38

**Table 3:** Testing errors for the three datasets. In the third column, we report the smallest errors achieved in [5] considering all Monte Carlo approximations implemented by the authors.

Dataset	pnnclass	Cucala et al.	Random Forest
Ripley	8.4%	8.4%	10.5%
Pima Indian	21.99%	20.9%	24.1%
Forensic Glass	28.04%	29%	24.3%

## Data Availability

The data used in the paper is available through the software library developed by the authors.
